# Supplementary material for: A novel approach for identifying and addressing case‐mix heterogeneity in individual participant data meta‐analysis
Source: Res Synth Methods. 2019 Dec 2;10(4):582–96. doi: 10.1002/jrsm.1382 (PMC6973268; doi:10.1002/jrsm.1382)
Supplement: Supplementary file 1 — Data S1. Supporting Information [file JRSM-10-582-s001.docx]

**SUPPORTING INFORMATION S1 –** Motivating examples

In the table S1, we present a brief example illustrating the issue of interaction depending on the effect measures and the effect of differences in case-mix. The outcome of interest is binary (response), and on the relative risk scale, an interaction occurs, since the relative risk differs between subgroups (small or large lesions). This is not the case on the odds ratio scale (both being equal to 2.67).

Two trials are conducted in two populations that differ in terms of case-mix, but not in terms of response rates and treatment effect among subgroups, all being the ones presented on the first two lines of the table S1. Sampling error occurs, but within what is expected. In both trials, the subgroups are balanced across randomization groups, so that no confounding may occur.

**Table S1. Illustrative example: description of true values and results of two hypothetical trials.** RR: relative risk; OR: odds ratio; Est.: estimate; 95% CI: 95% confidence interval.

|  | **Control** | | **Experimental** | | | **RR** | **OR** | |
| --- | --- | --- | --- | --- | --- | --- | --- | --- |
| **True values** | **Response rate** | | **Response rate** | | |  |  | |
| Small lesions | 60% | | 80% | | | 1.33 | 2.67 | |
| Large lesions | 20% | | 40% | | | 2.00 | 2.67 | |
|  |  | |  | | |  |  | |
|  |  | |  | | |  |  | |
| **Trials** | ***N*** | ***N* events** | ***N*** | | ***N* events** | **Est. (95% CI)** | **Est. (95% CI)** | |
| Trial 1 : population with 50% small and 50% large lesions; 400 patients randomized 1:1 | | | | | | | |  |
| Small lesions | 100 | 66 | 100 | 82 | | 1.24 (1.05–1.47) | 2.35 (1.22–4.53) | |
| Large lesions | 100 | 20 | 100 | 39 | | 1.95 (1.23–3.10) | 2.56 (1.36–4.82) | |
| Overall | 200 | 86 | 200 | 121 | | 1.41 (1.16–1.71) | 2.03 (1.36–3.03) | |
| Trial 2 : population with 25% small and 75% large lesions; 800 patients randomized 1:1 | | | | | | | |  |
| Small lesions | 100 | 64 | 100 | 81 | | 1.27 (1.06–1.51) | 2.40 (1.26–4.57) | |
| Large lesions | 300 | 131 | 300 | 54 | | 2.43 (1.85–3.19) | 3.53 (2.43–5.13) | |
| Overall | 400 | 118 | 400 | 212 | | 1.80 (1.50–2.15) | 2.69 (2.01–3.61) | |

**Figure S1. Random effects meta-analysis of the illustrative example, both on the relative risk (left panel) and odds ratio (right panel) scales.**

A random-effects meta-analysis as presented on the figure S1 identifies some heterogeneity on the relative risk scale (I^2^ = 71%), but much less on the odds ratio scale (I^2^ = 21%). When stratifying on the lesion size, effects within subgroups appear homogeneous, and close to the true values. Heterogeneity on the relative risk scale thus appears to be driven by between-subgroup differences, whereas no heterogeneity is left on the odds ratio scale. Note also that the summary estimate in terms of odds ratio differs between the unstratified and stratified analyses, whereas it remains nearly identical on the relative risk scale (though less precise in the stratified analysis). All those raise the question as to which populations the estimated intervention effects pertain.

**Figure S2. Random effects meta-analysis of the illustrative example stratified on the lesion size (subgroup analysis), both on the relative risk (left panel) and odds ratio (right panel) scales.**

With IPD, it is possible to use e.g. a logistic regression model to estimate the odds ratio adjusted for lesion size, both within each trial to be then meta-analyzed (two-stage approach) or using a single stage random effects model. Both are presented in the table S2 and yield the same results, virtually identical to the stratified analysis (figure S2).

**Table S2. Two-stage and one-stage IPD meta-analysis of the illustrative example.**

| **Study** | **Adjusted OR** |
| --- | --- |
| Trial 1 | 2.45 (1.56–3.87) |
| Trial 2 | 3.21 (2.33–4.44) |
|  |  |
| Two-stage random effects model | 2.94 (2.26–3.82) |
| Heterogeneity | I^2^ = 0%, τ^2^ = 0, *p* = 0.34 |
|  |  |
| One-stage random effects model | 2.94 (2.26–3.83) |
| Heterogeneity | I^2^ = 0%, τ^2^ = 0, *p* > 0.99 |

**SUPPORTING INFORMATION S2** – Derivation of the OCR-based and IPW-based estimators

**Derivation of OCR-based estimator**

Under the given assumptions, we have:

$P\left\{ Y\left( 1_{k} \right)=1 | S=j \right\}=E\left[ Y\left( 1_{k} \right)|S=j \right]$

$=E[E\left( Y\left( 1_{k} \right)\left| L,S=j \right) | S=j \right]$

$$=E[E\left( Y\left( 1_{k} \right)\left| X=1,L, S=j \right) | S=j \right]$$

$$=E[E\left( Y\left( 1_{k} \right)\left| X=1,L, S=k \right) | S=j \right]$$

$=E[E\left( Y\left| X=1,L, S=k \right) | S=j \right]$

Similarly, $P\left\{ Y\left( 0_{k} \right)=1 | S=j \right\}=E[E\left( Y\left| X=0,L, S=k \right) | S=j \right]$

**Derivation of IPW-based estimator**

$$P\left\{ Y\left( 1_{k} \right)=1 | S=j \right\} =\frac{1}{P\left( S=j \right)}E\left\{ I\left( S=j \right)Y\left( 1_{k} \right) \right\}$$

$$=\frac{1}{P\left( S=j \right)}E\left\{ E\left\{ I\left( S=j \right)Y\left( 1_{k} \right)| Y\left( 1_{k} \right),L \right\} \right\}$$

$$=\frac{1}{P\left( S=j \right)}E\left\{ Y\left( 1_{k} \right).E\left\{ I\left( S=j \right)|L \right\} \right\} as Y\left( 1_{k} \right)\perp S|L$$

$$=\frac{1}{P\left( S=j \right)}E\left\{ Y\left( 1_{k} \right).P\left( S=j | L \right) \right\}$$

$$=\frac{1}{P\left( S=j \right)}E\left\{ \frac{Y\left( 1_{k} \right).P\left( S=j | L \right)}{P\left( S=k | L \right)}E\left\{ I\left( S=k \right) | Y\left( 1_{k} \right),L \right\} \right\} as Y\left( 1_{k} \right)\perp S|L$$

$$=\frac{1}{P\left( S=j \right)}E\left\{ E\left\{ Y\left( 1_{k} \right).P\left( S=j | L \right)\frac{I\left( S=k \right)}{P\left( S=k | L \right)} | Y\left( 1_{k} \right),L \right\} \right\}$$

$$=\frac{1}{P\left( S=j \right)}E\left\{ Y\left( 1_{k} \right).P\left( S=j | L \right)\frac{I\left( S=k \right)}{P\left( S=k | L \right)} \right\}$$

$$=\frac{1}{P\left( S=j \right)}E\left\{ P\left( S=j | L \right).\frac{I\left( S=k \right)}{P\left( S=k | L \right)}Y\left( 1_{k} \right)\frac{E\left( X | L,S \right)}{P\left( X=1 | S \right)} \right\} as X\perp L|S$$

$$=\frac{1}{P\left( S=j \right)}E\left\{ P\left( S=j | L \right).\frac{I\left( S=k \right)}{P\left( S=k | L \right)}Y\left( 1_{k} \right)\frac{E\left( X | Y\left( 1_{k} \right),L,S \right)}{P\left( X=1 | S \right)} \right\} as Y\left( 1_{k} \right)\perp X|L,S$$

$$=\frac{1}{P\left( S=j \right)}E\left\{ E\left( P\left( S=j | L \right).\frac{I\left( S=k \right)}{P\left( S=k | L \right)}Y\left( 1_{k} \right).\frac{X}{P\left( X=1 | S \right)} | Y\left( 1_{k} \right),L,S \right) \right\}$$

$$=\frac{1}{P\left( S=j \right)}E\left\{ P\left( S=j | L \right).\frac{I\left( S=k \right)}{P\left( S=k | L \right)}Y\left( 1_{k} \right).\frac{X}{P\left( X=1 | S \right)} \right\}$$

$$=\frac{1}{P(S=j)}E\left\{ I\left( S=k \right).YX.\frac{P\left( S=j | L \right)}{P\left( S=k | L \right)}.\frac{1}{P\left( X=1 | S=k \right)} \right\}$$

And similarly,

$$P\left\{ Y\left( 0_{k} \right)=1 | S=j \right\}=\frac{1}{P(S=j)}E\left\{ I\left( S=k \right).Y(1-X).\frac{P\left( S=j | L \right)}{P\left( S=k | L \right)}.\frac{1}{P\left( X=0 | S=k \right)} \right\}$$

Besides, it can be shown that:

$$\frac{1}{P(S=j)}E\left\{ I\left( S=k \right).I(X=x).\frac{P\left( S=j | L \right)}{P\left( S=k | L \right)}.\frac{1}{P\left( X=x | S=k \right)} \right\}=1$$

$P\left\{ Y\left( 1_{k} \right)=1 | S=j \right\}$therefore can be rewritten as:

$$P\left\{ Y\left( x_{k} \right)=1 | S=j \right\}=\frac{E\left\{ I\left( S=k \right).Y.I(X=x).\frac{P\left( S=j | L \right)}{P\left( S=k | L \right)} \right\}}{E\left\{ I\left( S=k \right).I(X=x).\frac{P\left( S=j | L \right)}{P\left( S=k | L \right)} \right\}}$$

**SUPPORTING INFORMATION S3 – Odds ratio scale**

$OR\left( j,k \right)$ can be defined as :

$$OR\left( j,k \right)=\frac{\frac{P\{Y(1_{k})=1|S=j\}}{1-P\{Y(1_{k})=1|S=j\}}}{\frac{P\{Y(0_{k})=1|S=j\}}{1-P\{Y(1_{k})=1|S=j\}}}$$

Under the 4 assumptions given in section 3.1, $OR\left( j,k \right)$can be rewritten as:

$$OR\left( j,k \right)=\frac{\frac{E[E\left( Y\left| X=1,L, S=k \right) | S=j \right]}{1-E[E\left( Y\left| X=1,L, S=k \right) | S=j \right]}}{\frac{E[E\left( Y\left| X=0,L, S=k \right) | S=j \right]}{1-E[E\left( Y\left| X=1,L, S=k \right) | S=j \right]}}$$

Under the model (1) in section 2, a straightforward estimator of $OR\left( j,k \right)$ can be obtained by using outcome regression:

$$\hat{OR}\left( j,k \right)=\frac{\frac{\sum_{i} I\left( S_{i}=j \right)expit\left\{ \hat{\beta}_{0k}+\hat{\beta}_{1k}+\hat{\beta}_{2k}L_{i} \right\}}{n_{j}-\sum_{i} I\left( S_{i}=j \right)expit\left\{ \hat{\beta}_{0k}+\hat{\beta}_{1k}+\hat{\beta}_{2k}L_{i} \right\}}}{\frac{\sum_{i} I\left( S_{i}=j \right)expit\left\{ \hat{\beta}_{0k}+\hat{\beta}_{2k}L_{i} \right\}}{n_{j}-\sum_{i} I\left( S_{i}=j \right)expit\left\{ \hat{\beta}_{0k}+\hat{\beta}_{2k}L_{i} \right\}}}$$

where $n_{j}$ stands for the number of patients in trial $j$ and $\hat{\beta}_{0k}, \hat{\beta}_{1k}, \hat{\beta}_{2k}$are estimates obtained by fitting the above model to the data from trial $k.$

Another approach to estimate $OR\left( j,k \right)$ is to use inverse probability weighting:

$$\hat{OR}\left( j,k \right)=\frac{\frac{\sum_{i}^{n} I\left( S_{i}=k \right)Y_{i}X_{i}\frac{\hat{P}\left( S_{i}=j | L_{i} \right)}{\hat{P}\left( S_{i}=k | L_{i} \right)}}{\sum_{1}^{n} I(X_{i}=1)I\left( S_{i}=k \right)-\sum_{i}^{n} I\left( S_{i}=k \right)Y_{i}X_{i}\frac{\hat{P}\left( S_{i}=j | L_{i} \right)}{\hat{P}\left( S_{i}=k | L_{i} \right)}}}{\frac{\sum_{i}^{n} I\left( S_{i}=k \right)Y_{i}\left( 1-X_{i} \right)\frac{\hat{P}\left( S_{i}=j | L_{i} \right)}{\hat{P}\left( S_{i}=k | L_{i} \right)}}{\sum_{1}^{n} I(X_{i}=0)I\left( S_{i}=k \right)-\sum_{i}^{n} I\left( S_{i}=k \right)Y_{i}\left( 1-X_{i} \right)\frac{\hat{P}\left( S_{i}=j | L_{i} \right)}{\hat{P}\left( S_{i}=k | L_{i} \right)}}}$$

**SUPPORTING INFORMATION S4 –** Baseline characteristics (6 trials) in the vitamin D dataset

| **Trial** | **1** | **2** | **3** | **4** | **5** | **6** |
| --- | --- | --- | --- | --- | --- | --- |
| **N** | 162 | 164 | 250 | 240 | 240 | 34 |
| **Randomisation** | 1 : 1 | 1 : 1 | 1:1 | 1 : 1 | 1 : 1 | 1 : 1 |
| **Sex**  Female, % | 79.01 | 0.0 | 56.4 | 40.0 | 65.8 | 58.82 |
| NA | 0 | 0 | 0 | 0 | 0 | 0 |
| **Age at baseline** | | | | | | |
| Min | 21.4 | 18 | 16 | 40 | 21.4 | 18 |
| Q1 | 49.88 | 19 | 37 | 60 | 60.2 | 21.25 |
| Median | 58.80 | 19 | 48 | 64 | 68.5 | 27 |
| Mean | 57.87 | 19.07 | 47.87 | 64.69 | 67.09 | 32.21 |
| Q3 | 68.12 | 19 | 59 | 70 | 75.25 | 45.25 |
| Max | 80.60 | 21 | 78 | 85 | 94 | 52 |
| NA | 0 | 0 | 0 | 0 | 0 | 0 |
| **BMI at baseline** | | | | | | |
| Min | 18.43 | 17.18 | 17.72 | 16.02 | 17.34 | 18.36 |
| Q1 | 23.19 | 21.49 | 23.35 | 22.55 | 24.90 | 22.20 |
| Median | 26.25 | 23.31 | 26.33 | 26.80 | 27.72 | 24.87 |
| Mean | 27.34 | 23.42 | 27.61 | 27.51 | 29.28 | 25.28 |
| Q3 | 29.41 | 25.32 | 30.10 | 31.62 | 32.44 | 27.23 |
| Max | 117.15 | 31.71 | 56.77 | 48.71 | 58.69 | 46.49 |
| NA | 0 | 12 | 0 | 0 | 0 | 0 |
| **Influenza vaccination**  Yes (%) | 58.02 | 74.39 | 86.0 | 92.08 | 66.67 | 41.94 |
| NA | 0 | 0 | 0 | 0 | 0 | 3 |
| **Pneumococcal vaccination**  Yes (%) | - | - | 32.4 | 64.17 | 37.92 | 22.73 |
| NA | 162 | 164 | 0 | 0 | 0 | 12 |
| **Vitamin D concentration at baseline** | |  |  |  |  |  |
| Min | 16.0 | 41.9 | 0.0 | 0.0 | 0.0 | 32 |
| Q1 | 46.1 | 62.4 | 31.0 | 27.0 | 24.75 | 50 |
| Median | 60.5 | 73.4 | 45.5 | 42.5 | 40.50 | 68 |
| Mean | 63.68 | 75.88 | 49.56 | 46.1 | 42.91 | 67.94 |
| Q3 | 76.4 | 88.8 | 62.75 | 61.0 | 56.0 | 78 |
| Max | 156 | 129.0 | 139.0 | 160.0 | 128.0 | 132 |
| NA | 12 | 91 | 0 | 0 | 0 | 1 |

*Trial 1 : Li-Ng, 2009; trial 2: Laaksi 2010; trial 3 : Martineau, 2015a; trial 4: Martineau, 2015b; trial 5: Martineau, 2015c; trial 6: Simpson, 2015*

**SUPPORTING INFORMATION S5 –** Data analysis: p-values of the tests for heterogeneity assessment

| **Heterogeneity** | **Contrast (log scale)** | **OCR** | **IPW** |
| --- | --- | --- | --- |
| **Beyond case-mix** | $OR(1,1)=OR(1,2)=OR(1,3)=OR(1,4)$ | 0.55 | 0.69 |
|  | $OR(2,1)=OR(2,2)=OR(2,3)=OR(2,4)$ | 0.55 | 0.43 |
|  | $OR(3,1)=OR(3,2)=OR(3,3)=OR(3,4)$ | 0.55 | 0.55 |
|  | $OR(4,1)=OR(4,2)=OR(4,3)=OR(4,4)$ | 0.54 | 0.86 |
|  |  |  |  |
| **Case-mix** | $OR(1,1)=OR(2,1)=OR(3,1)=OR(4,1)$ | 0.99 | 0.28 |
|  | $OR(1,2)=OR(2,2)=OR(3,2)=OR(4,2)$ | 0.93 | 0.23 |
|  | $OR(1,3)=OR(2,3)=OR(3,3)=OR(4,3)$ | 1.00 | 0.75 |
|  | $OR(1,4)=OR(2,4)=OR(3,4)=OR(4,4)$ | 0.83 | 0.40 |

**SUPPORTING INFORMATION S6** – Weight distribution after truncation and variance of the IPW estimates for $OR(j,k)’s$

**6.1 Weight distribution after truncation**

*In one cell, from top to bottom: minimum value, 1^st^ quantile, median, mean, 3^rd^ quantile, maximum value of the weight distribution used for the IPW-based estimator of* $OR(j,k)$

|  | **k = 1** | **k = 2** | **k = 3** | **k = 4** |
| --- | --- | --- | --- | --- |
| **j = 1** | - | 0.00  0.30  0.75  1.51  1.90  7.06 | 0.01  0.23  0.52  1.28  1.33  6.85 | 0.00  0.26  0.65  2.99  1.83  26.0 |
| **j = 2** | 0.02  0.53  1.33  3.40  3.29  20.0 | - | 0.00  0.27  0.75  2.94  2.48  21.9 | 0.00  0.19  0.79  25.4  4.61  330.97 |
| **j = 3** | 0.01  0.75  1.94  3.52  4.39  15.6 | 0.00  0.40  1.33  3.53  3.71  20.4 | - | 0.02  0.43  1.19  4.17  3.60  28.5 |
| **j = 4** | 0.00  0.55  1.55  2.76  3.84  11.68 | 0.00  0.22  1.27  4.95  5.16  31.7 | 0.00  0.28  0.84  1.61  2.33  6.76 | - |

**6.2 Variance of the IPW estimate for** $\mathbf{log}\boldsymbol{OR(j,k)}$

|  | **k = 1** | **k = 2** | **k = 3** | **k = 4** |
| --- | --- | --- | --- | --- |
| **j = 1** | 0.19 | 0.58 | 1.01 | 4.25 |
| **j = 2** | 0.80 | 0.28 | 0.24 | 3.54 |
| **j = 3** | 0.43 | 0.68 | 0.19 | 0.33 |
| **j = 4** | 0.24 | 1.09 | 0.37 | 0.17 |
